# Supplementary material for: Chitosan–Imidazolium Core–Shell Nanoparticles of Gd-Mn-Mo Polyoxometalate as Novel Potential MRI Nano-Agent for Breast Cancer Detection
Source: Micromachines (Basel). 2023 Mar 27;14(4):741. doi: 10.3390/mi14040741 (PMC10143193; doi:10.3390/mi14040741)
Supplement: Supplementary file 1 [file micromachines-14-00741-s001.zip › micromachines-2283924-supplementary.pdf]

Chitosan-Imidazolium Core-Shell Nanoparticles of Gd-Mn-Mo Polyoxometalate as Novel Potential MRI Nano-agent for Breast Cancer Detection

Fahimeh Aminolroayaei [1], Daryoush Shahbazi-Gahrouei [2,*], Mahboubeh Rostami [3,*], Seyed Hossein Hejazi [4] and Amin Farzadniya [5]

[1] Department of Medical Physics, School of Medicine, Isfahan University of Medical Sciences, Isfahan, Iran
[2] Department of Medical Physics, School of Medicine, Isfahan University of Medical Sciences, Isfahan, Iran
[3] Novel Drug Delivery Systems Research Centre and Department of Medicinal Chemistry, School of Pharmacy and Pharmaceutical Sciences, Isfahan University of Medical Sciences, Isfahan, Iran
[4] Department of Parasitology and Mycology, School of Medicine, Isfahan University of Medical Sciences, Isfahan, Iran
[5] Department of Radiology, Askarieh Hospital, Isfahan, Iran
* Correspondence: shahbazi@med.mui.ac.ir (D.S.-G.); m.rostami@pharm.mui.ac.ir (M.R.); Tel.: +98-31-37929095 (D.S.-G.); +98-31-37927107 (M.R.)

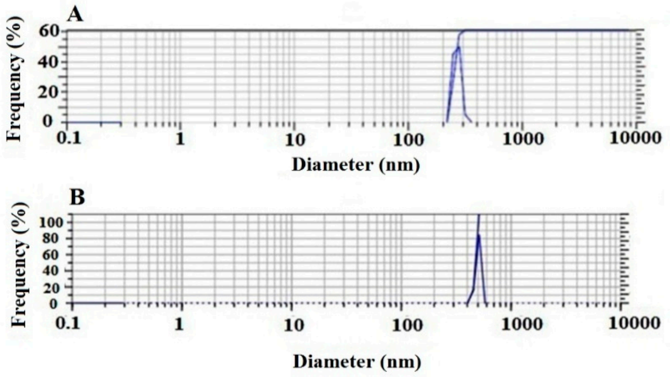

Figure S1. Dynamic light scattering (DLS) results of: A) Gd-Mn-Mo POM and B) Gd-Mn-Mo POM@Cs-Im.

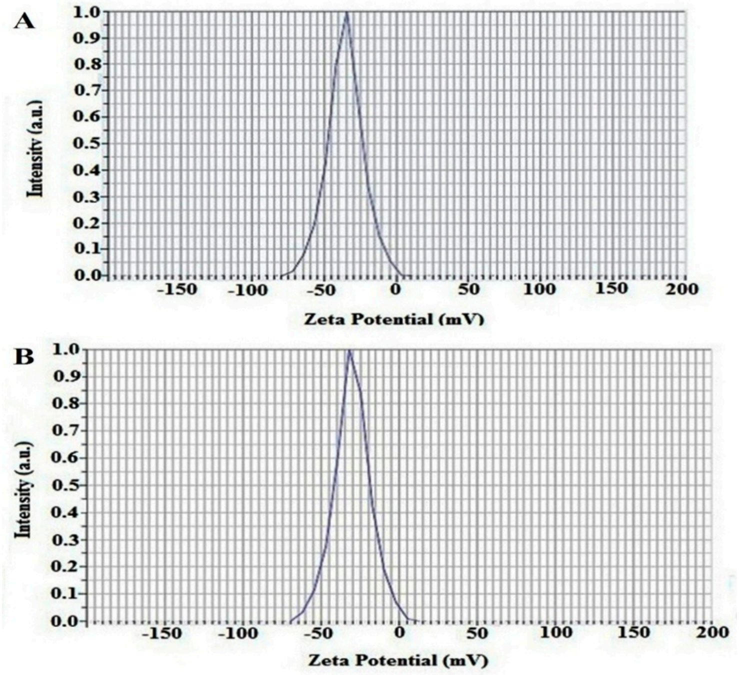

Figure S2. Zeta potential results of: A) Gd-Mn-Mo POM and B) Gd-Mn-Mo POM@Cs-Im.

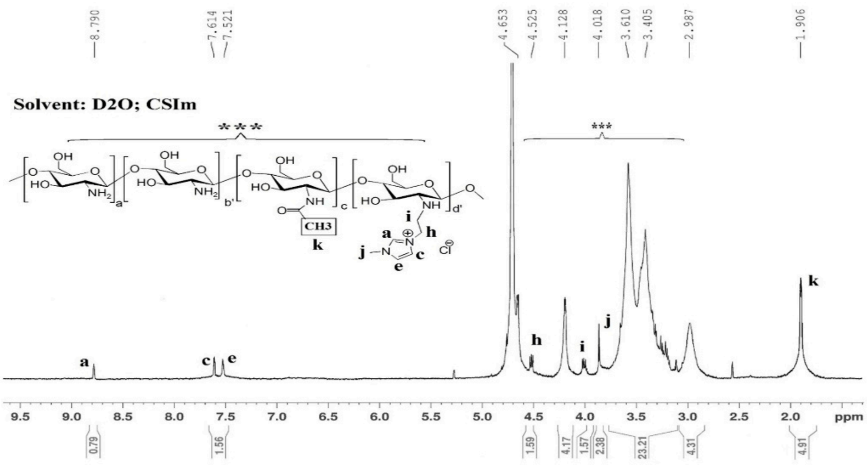

Figure S3. HNMR results of shell: Signals around 7.5 and 8.7 ppm have approved the presence of imidazolium aromatic features of CSIm. That is in agreement with the previous study [20].

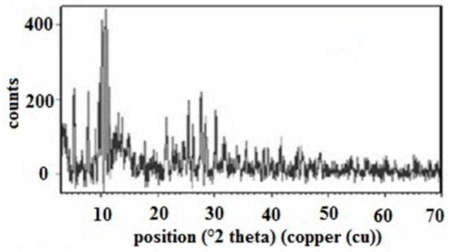

Figure S4. XRD results of Gd-Mn-Mo POM recorded at room temperature in 2θ range of 3–70°.

Reference

20. Mahvash, S.; Zavareh, V.A.; Taymouri, S.; Ramezani-Aliakbari, M.; Dousti, F.; Mirian, M.; Rostami, M. Hybrid Nanocomposite of Imidazolium Based Chitosan and Anderson-type Manganese Polyoxomolybdate for Boosting Drug Delivery Against Breast Cancer. Research Square; 2021. DOI: 10.21203/rs.3.rs-729081/v1.
